# What Influences the Sustainable Food Consumption Behaviours of University Students? A Systematic Review

**DOI:** 10.3389/ijph.2021.1604149

**Published:** 2021-09-07

**Authors:** Lucía Aguirre Sánchez, Zayne M. Roa-Díaz, Magda Gamba, Giorgia Grisotto, Ana Maria Moreno Londoño, Blanca Patricia Mantilla-Uribe, Alba Yaneth Rincón Méndez, Mónica Ballesteros, Doris Kopp-Heim, Beatrice Minder, L. Suzanne Suggs, Oscar H. Franco

**Affiliations:** ^1^ Institute of Public Health (IPH), Università della Svizzera italiana, Lugano, Switzerland; ^2^ Institute of Communication and Public Policy (ICPP), Università della Svizzera italiana, Lugano, Switzerland; ^3^ Institute of Social and Preventive Medicine (ISPM), University of Bern, Bern, Switzerland; ^4^ Graduate School for Health Sciences, University of Bern, Bern, Switzerland; ^5^ Harvard T.H. Chan School of Public Health, Boston, MA, United States; ^6^ Independent Researcher, Lugano, Switzerland; ^7^ Instituto Proinapsa, Universidad Industrial de Santander, Bucaramanga, Colombia; ^8^ Centro de Investigación Biomédica en Epidemiología y Red de Salud Pública, Instituto de Salud Carlos III (ISCIII), Madrid, Spain; ^9^ Public Health and Primary Care Library, University of Bern, Bern, Switzerland; ^10^ Swiss School of Public Health (SSPH+), Zurich, Switzerland

**Keywords:** sustainable food consumption, sustainable diets, pro-environmental behaviour, health behaviour, university students, young adults, young people, systematic review

## Abstract

**Objectives:** Global environmental challenges demand sustainable behaviours and policies to protect human and planetary health. We aimed to summarize the evidence about the factors related to Sustainable Food Consumption (SFC) behaviours of university students, and to propose an operational categorization of SFC behaviours.

**Methods:** Seven databases were searched for observational studies evaluating Sustainable Food Consumption (SFC) among university students and that reported at least one behavioural outcome measure. Qualitative synthesis was conducted, and PRISMA guidelines for reporting were followed.

**Results:** Out of 4,479 unique references identified, 40 studies were selected. All studies examined personal factors, while 11 out of 40 also measured social or situational factors. Except for food waste, females had higher levels of SFC behaviours, but situational factors moderated this association. Knowledge and attitudes showed mixed results. Overall, sustainable food consumers reported healthier lifestyles.

**Conclusions:** Healthy lifestyle of sustainable food consumers suggests possible synergies between human health and sustainability in terms of motivations for food choice. Moderation effects of social and situational factors on personal factors reveal opportunities to design and examine the effects of choice architecture interventions.

## Introduction

Food connects human and planetary health. Diet-related factors are among the top contributors to the global burden of disease [1], and the food sector is the leading cause of environmental change, contributing to 19–29% of the global Greenhouse gas (GHG) emissions [2]. Climate change drives adverse effects back into human health [3], affecting food availability, the nutritional contents of foods, and putting populations at risk of nutritional deficiencies [4].

FAO defines sustainable diets as those with “low environmental impacts which contribute to food and nutrition security and to healthy life for present and future generations […]” [5], in line with earlier definitions of sustainable consumption [6]. The EAT-Lancet Commission proposes a healthy diet from sustainable food systems [3], identifying three main spheres for food system transformation: improvements in production, widespread change in dietary patterns, and waste reduction. However, to date, there is no operational and widely accepted definition of sustainable food consumption behaviours, and the factors associated remain unclear.

While health and environmental co-benefits of sustainable diets have been reported in the literature [7–10], from the consumer perspective, sustainable food consumption may pose a tension between individual and collective interests, adding a pro-social aspect to food consumption. Therefore, behavioural approaches are needed to understand what drives the adoption of healthier and more sustainable eating behaviours, especially those with lower environmental impact [3, 11]. However, research about behavioural aspects of sustainable food consumption is considered scarce compared to the extensive body of evidence on the adverse environmental and health impact of eating behaviours [12–14].

University students, in particular, are more willing to adopt changes in their eating behaviours, and are more environmentally conscious than older generations [15]. Universities are the organizations where studies on behaviour and consumption are most frequently conducted, with estimations of up to 80% of the literature in this field is based on student samples [16].

University students engage in unhealthy eating behaviours [17], which has yielded a vast body of literature on the importance of healthy diets among this population. The adherence to food consumption behaviours that are healthy and also sustainable has gained some attention [18–20]. Hence, we aimed to systematically summarize the evidence regarding the underlying factors that can determine or constrain sustainable food consumption among university students and propose an operational categorization of Sustainable Food Consumption (SFC) behaviours.

## Methods

This systematic review was conducted following the guide proposed by Muka and colleagues [21] and the PRISMA guidelines for reporting [22]. The protocol is registered in PROSPERO: CRD42021233347.

### Data Source and Search Strategy

The search strategy (See search strategies in Supplementary Material) was developed by the authors, including two librarians. The search was limited to human studies and peer-review publications. The search terms included synonyms of sustainable food consumption and specific behaviours based on relevant literature, on diets and food systems with lower environmental impact [3, 23]. Medline, Embase, PsycInfo, Web of Science, Scopus, LILACS databases and Google Scholar were searched to identify relevant articles from inception until 27 January 2021 without language or geographic restrictions [3, 23, 24]. Backward reference search was conducted on each of the studies selected from the database search. Expert input and a manual search in relevant journals were also used (See Search Strategies in Supplementary Material).

### Selection Criteria

Studies were included if they: were conducted with university students; were observational (e.g., cross-sectional); presented behavioural outcome measures of SFC, and identified factors associated with SFC. Our operational definition of Sustainable Food Consumption includes both dietary patterns and other consumer behaviours related to how food is produced, processed, transported, managed and wasted. Building on Garnett et al 2014 [23], the outcome also includes behaviours such as choosing foods with less energy-intensive transport modes, such as local and seasonal products, meat eaten in moderate quantities, dairy products or alternatives eaten in moderation, and tap water in preference to other beverages.

Studies were excluded if participants reported comorbidities or were post-doctoral researchers, evaluated the efficacy or effectiveness of interventions focused on farming, agriculture, or other food production-related behaviours, or assessed behavioural intentions, attitudes, and willingness but not actual behaviours. Cost-effectiveness studies, case reports, letters to the editor, conference proceedings, systematic reviews, or meta-analyses were also excluded.

### Screening and Study Selection

Pairs of screeners independently reviewed titles and abstracts of the retrieved references. Overlapping references were included for full-text screening. Inclusion disagreements were solved initially by the reviewers and persistent disagreements were solved upon consultation with a third reviewer.

### Data Analysis and Synthesis of Results

A tailored data extraction form was developed and piloted for this study. The form included identifiers, general characteristics of the study and participants, and results (See Supplementary Material). Qualitative analysis of the reviewed articles was conducted following deductive categorization of behavioural outcomes, data reduction, and narrative synthesis of related factors, as associations, correlations and descriptive group comparisons. The proposed behavioural categories were built deductively from relevant literature, while the target behaviours were extracted from the measurement instruments reported in selected articles. Given the diversity of measurement approaches, quantitative meta-analysis was not performed.

### Quality Assessment

Two reviewers independently assessed the quality of included studies using the Newcastle–Ottawa Scale [25] (NOS) for cross-sectional studies; disagreements were solved by consensus. NOS was developed for non-randomized and observational studies and assessed quality in three broad categories: selection of study groups/participants, comparability of the study groups/participants, and the assessment of outcome of interest. Quality was assessed on a 10-point scale and classified as good (10–9 points), moderate (8–6 points), and low quality (≤5 points). All studies were included in the analysis, independently of NOS score.

## Results

### Study Selection

**FIGURE 1:**
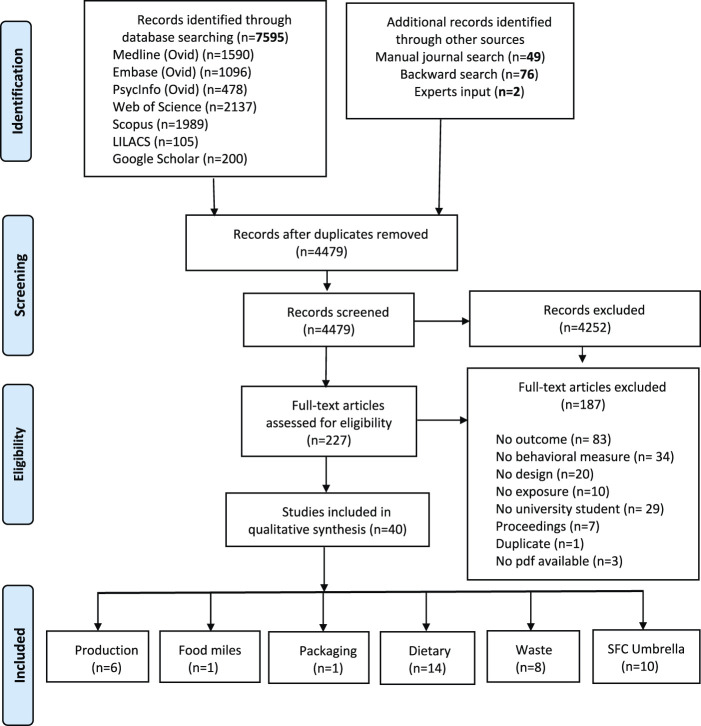
Preferred Reporting Items for Systematic reviews and Meta-Analyses (PRISMA) flowchart for selected articles. (What influences the sustainable food consumption behaviours of university students? A systematic review, several countries, 2021).

We identified 4,479 unique references, of which 227 were selected to be screened in full text. Of those, 40 studies comprising 27,946 participants met the selection criteria (See Figure 1). A summary of included papers is presented in Table 1.

**TABLE 1:** Articles included in the review. (What Influences the Sustainable Food Consumption Behaviours of University Students? A Systematic Review, Several countries, 2021).

Author, year, country	n^a^, (% of women), age	Behavioural outcome	Examined factors (in addition to demographics^b^)	Key results (significant associations or significant group differences)	Quality (NOS)^c^
Akbar et al., 2019, Pakistan	*n* = 221, (33.5), NR	Organic Food Buying Behaviour	Green Perceived Value (GPV) constructs (functional value, social value, emotional value, and conditional value), purchase intention, food neophobia.	Food neophobia moderates the relationship of purchase intention and behaviour.	Moderate
Dahm et al., 2009, United States	*n* = 443, (55.8), *M* = 21.6, SD 5.01	Organic food consumption and purchase	Awareness (knowledge) and attitudes toward organic foods, and attitudes and behaviours regarding other eco-friendly practices.	Attitude predicted purchase and consumption of organic foods on campus.	Low
Giampietri et al., 2020., Italy	*n* = 223, (47.1), *M* = 22.45 SD 2.5	Organic food consumption	Individual risk attitude	More risk averse individuals eat organic food frequently. Trust and social norms were linked to organic food consumption.	Low
Hamilton and Hekmat, 2018, Canada	*n* = 426, (NR), NR	Organic food consumption	Knowledge and attitudes.	Attitudes (safety of organic food, the nutrition value of organic food, the perception that organic food is fresher and better in taste and the perception that organic food is better for animal welfare and the environment) were significantly associated with the frequency of consumption. Perceived safety was highly correlated with organic food consumption.	Low
McReynolds et al., 2017., United States	*n* = 238, (40), *M* = 22.4 SD 6.5	Organic food consumption and purchase	Knowledge and perceptions about organic food, demographic and socioeconomic characteristics	There was a significant correlation between students’ knowledge and behaviour. Having experience growing fruits or vegetables had the greatest impact.	Low
Zámková and M. Prokop, 2013, Czech Republic	NR	Organic food consumption	Exposure to organic food advertisement.	Women bought organic food more frequently than men. Main reason of disinterest in buying organic is the price, distrust and not believing that organic food is better than conventional food. Advertising for organic food did not affect purchase.	Low
Fernandez-Ferrin et al., 2017, Spain	*n* = 195, (55.4), *M* = 21.34 SD Not applicable	Locally produced food products	Local identity, brand valuation, and moderating effect of perceived availability.	Perceived availability condition the promoting effect of local identity on purchase of local tomato sauce and local rice. Perceived availability does not moderate purchase of local mineral water and traditional cake.	Low
Diez et al., 2018, Spain	*n* = 632, (61.2), NR	Tap water and bottled water consumption frequency	Perceptions about bottled and tap water.	Students presented the highest proportions of consumption of more than 6 bottles of water per week. Beliefs (e.g., “I trust tap water’s quality,” “If I drink tap water, I am contributing less plastic to landfills”) had statistical differences between low consumption group (0 bottles per week) and high bottled water users (≥6 bottles per week). No associations with gender.	Moderate
Barros et al., 2020, Brazil	*n* = 1841, (54.8), NR	Prevalence of vegetarian diet	Lifestyle characteristics.	Males had less odds of being vegetarians. Those who reported prejudicial alcohol use were almost twice as likely to adopt a vegetarian diet.	Moderate
Forestell et al., 2012, United States	*n* = 240, (100), M = 19.28	Vegetarian, pesco-, semi-vegetarian, and flexitarian compared to omnivores	Food restraint, lifestyle (e.g., drinking alcohol, smoking), personality inventory, variety seeking, food neophobia, general neophobia, food choice, sensory appeal, price, familiarity, mood, ethical concern, eating attitudes.	Vegetarians and pesco-vegetarians were more open to new experiences, variety seeking, and less food neophobic than regular omnivores. Semi-vegetarians and flexitarians were more restrained than omnivores.	Low
Forleo et al., 2017, Italy	*n* = 548, (67.1), *M* = 25.05	Adherence to new Italian Mediterranean food pyramid	Living with parents or away, eating at home or away, BMI, pro-environmental behaviours, knowledge on daily caloric needs, physical activity (sports).	Six clusters were identified. Cluster five (26% of the sample), the least compliant to Mediterranean Pyramid, showed an above average consumption of meat and processed meat products, younger students, a higher percentage of females and students living with parents.	Low
Izmirli and Phillips, 2011, Multiple countries	*n* = 3,433, (NR), NR	Consumption of animal products	Attitudes towards animals, and perceived importance of world issues.	Students avoiding some meats cited the environment as the most important reason, and then health, whereas most vegetarian students gave their health as the main reason. Vegans had greater concern for animal welfare.	Low
Kawasaki et al., 2021, Japan	*n* = 215, (100), *M* = 20	Healthful plant-based diet	Mindful eating	Higher scores for healthful plant-based diet were correlated with higher “health of the planet” and “awareness and appreciation for food” sub-scores. “Non-judgmental awareness” was correlated with a low intake of healthful plant-based foods.	Low
Llanaj and Hanley-Cook, 2020., Albania	*n* = 289, (87.19), NR	Adherence to EAT–Lancet diet	Anthropometric measurements, dietary intake, dietary cost and eating out of home	EAT-lancet diet adherence was very low. No associations found.	Moderate
Pocol et al., 2020, Romania, Bulgaria and Moldova.	*n* = 2,378, (NR), NR	Adherence to mixed or vegetarian diets.	Residence, weight.	Mixed diet was slightly higher among men. Semi-vegetarian, ovo-lacto-vegetarian, and lacto-vegetarian diets, was slightly higher among women. Mixed diet decreases with age, while semi-vegetarian and ovo-lacto-vegetarian diets, increases slightly with age.	Low
Menozzi et al 2017, Italy	*n* = 231, (61.9), *M* = 23.6 SD 3.8	Tasting an edible insect food product	Attitudes, subjective norms, perceived behavioural control (PBC), intention, topic of study.	Students enrolled in social sciences-related were less likely to taste the insect-based food product compared to students in food and environmental sciences-related. intention is the main predictor of the behaviour, followed by perceived behavioural control.	Low
Olfert et al., 2020., United States	*n* = 1,078, (66,6), NR	Adherence to vegetarian diet	Perceptions of campus environment, waist, and hip circumference, fruit and vegetable intake, fat intake, stress, eating attitudes, physical activity, sleep quality.	Vegetarians had higher stress, consumed significantly more servings of fruits and/or vegetables per day and obtained a lower percentage of their daily caloric intake from fats than nonvegetarians. Vegetarians had similar mean BMI as nonvegetarians.	Low
Ruby et al. 2016, Argentina, Brazil, France, United States	*n* = 1,695, (65.5), *M* = 22 SD 2.53	Beef consumption	Attitudes toward beef, and toward vegetarians.	Men consumed beef significantly more often than women. Consumption was significantly highest in Brazil, followed by Argentina, then the USA, and finally France. Men ate beef significantly more frequently than did women in Brazil, and the United States, but not in Argentina.	Low
Smith et al., 2000, United States	NR	Adherence to vegetarian, vs. weight loss diet	Reasons for discontinuing diets	Vegetarian group remained on their diet for more than 1 year, whereas the majority of the Weight-Loss participants followed their diet for 1–3 months. Strictness of diets did not differ. Main reasons cited for stopping the vegetarian diet were “missed eating (meat),” “inconvenience,” and “did not get adequate nutrients.”	Low
Spencer et al., 2007, United States	NR	Self-reported vegetarian diet	Health-related outcomes	Vegetarians were more likely to eat more fruits and vegetables, be women, be Hindu, Buddhist, or Seventh Day Adventist, be politically liberal, have a BMI ≤ 25	Low
Suleiman et al., 2009, Jordan	*n* = 1,500, (65.4), *M* = 20.3 Range 17–28	Prevalence vegetarian diet	Selected demographic and lifestyle characteristics.	The vegetarian group consisted mainly of women, aged between 17 and 20 years, with low income, non-smokers, physically active, using vitamin and mineral supplements and having a normal BMI.	Moderate
Vizcaino et al., 2020, United States	*n* = 99, (70), Median = 18 Interquartile range 18–19	Adherence to a plant-based diet	Self-regulatory system, variety of motivations.	Successful adherents had higher levels of value, self-efficacy, planning/stimulus control and positive affect, were seventeen times more likely to report “To manage or treat a medical condition” and were 94% less likely to report ‘To maintain and/or improve my health’ as motivation.	Moderate
Al-Domi H, 2011, Jordan	*n* = 600, (37), *M* = 21.5 SD 2.85	Plate waste	Demographic and socioeconomic characteristics.	No differences between the food plate wasted between women and men, except for meat wasted, women waste slightly more than men. Food waste was low in general.	Low
Alattar et al., 2020, United States	*n* = 495, (54), *M* = 21 Range 18–58	Food waste diversion behaviours	Food management skills, food waste attitudes/emotions, perception of cost, food waste knowledge, general sustainability beliefs, perception of personal impact.	The composting index was negatively correlated with food waste diversion intent, but attitudes toward composting were still positively correlated.	Low
Lorenz et al., 2017, Germany	*n* = 238, (48), NR	Leftover behaviour (Food waste)	Personal (Attitudes, PBC, subjective norms, Intention, Personal Norms), social (presence of others), and environmental/situational factors (palatability, portion size, and time pressure)	Perceptions of food (portions size and palatability) was related to food leftovers. For participants under time pressure, gender (being female) becomes a significant determinant for leftover behaviour. Time pressure was not a direct environmental determinant of leftovers.	Low
Lorenz et al., 2018, Germany	*n* = 384, (47), *M* = 24.3	Visually estimated food leftovers	Beliefs (constructs: environment, self-interest, and resources), general attitude (towards the behaviour) and behavioural intention	Larger perceived portion size related to increased leftovers, and more positive taste evaluation related to lower leftovers. Both situational variables are significantly correlated with the self-interest but not with pro-environmental or resource efficiency beliefs.	Moderate
Mondejar-Jimenez et al., 2017, Spain, Italy	*n* = 380, (58), *M* = 20.62 SD 2.62	Positive behaviour towards food waste, and proportion food wasted	Concern about food waste, moral attitude, subjective norms, perceived behavioural control, marketing/sale addiction, intention	Subjective norms and perceived behavioural control promote the positive behaviour. Marketing/sale strategies “addiction” decreased the positive behaviour. The strongest positive significant total effect on behaviour comes from subjective norms.	Low
Morata Verdugo et al., 2020, Spain	*n* = 49, (75,5), *M* = 22.6 SEM ± 6.0	Food waste at home (leftovers)	Eating habits and level of physical activity.	Food waste was slightly higher among women. Lunch generated more wasted food than dinners.	Low
Principato et al, 2015, Italy	*n* = 233, (39), NR Range 19–28	Food waste reduction behaviours	Knowledge, attitudes (level of concern).	Food waste reduction behaviour was negatively associated with higher levels of concern about “the risk of eating unsafe food because is no longer fresh,” belief that “only 10% of the food purchased gets thrown away” (compared to those who believe that higher percentages of food get thrown away,” and believing that “packaging of the food thrown in the trash is a larger environmental problem than food waste.”	Low
Wu, et al., 2019, China	*n* = 551, (46.1), NR	Plate waste	Attitudes, perceived behavioural control, subjective norms, canteens characteristics (e.g., food not tasty, too much food provided), factors related to food waste avoidance (e.g., save money, felling of guilt)	A perception that avoiding food waste is difficult, and higher living expenses were factors promoting food waste. Subjective norms, attitudes, gender, and major had insignificant impacts. Male students wasted significantly less staple foods than female students.	Moderate
Anh et al., 2019, Vietnam	*n* = 791, (NR), NR	Sustainable consumption behaviour in food	Environmental awareness and action, economical and effective options, and sustainable buying options.	The construct of “Environmental awareness and action” (I cook in an energy-efficient way, I avoid eating convenience food because of plastic waste, I use containers instead of plastic wraps/bags, I sort the inorganic or organic waste before throwing into the trash) had the strongest positive impact on the studied outcome.	Low
Anh et al., 2020, Vietnam	*n* = 791, (59), NR	Sustainable consumption behaviour in food and drink	Gender, religion, academic year, love relationship, residence status.	Being in a relationship increased the probability of sustainable consumption behaviour.	Low
Campbell-Arvai, 2015, United States	*n* = 320, (52.5), NR	Food-related environmental behaviours	Demographic and socioeconomic characteristics, value orientation, pro-environmental worldview (NEP – New environmental paradigm scale), and food-related environmental beliefs	Higher BVO (Biospheric value orientation) and environmental belief scores were associated with higher environmental behaviour scores. NEP scores (New environmental paradigm), when controlling for BVO, environmental beliefs and gender, did not make a significant contribution to the model. Males had lower environmental behaviour scores than females.	Low
Dopelt et al., 2019, Israel	*n* = 361, (75), *M* = 29 SD 8.6	Pro-environmental behaviours (food related)	Knowledge, attitudes.	Women had more pro-environmental behaviour than men. Attitudes were the best predictor of pro-environmental food related behaviour. Lack of knowledge on environmental impact of food consumption was negatively correlated to outcome.	Low
Kamenidou et al., 2019., Greece	*n* = 252, (54.8), NR	Sustainable food consumption behaviour	Social norms, ecological purchase behaviour, and clusters based on demographic characteristics.	Two students’ segments were identified based on Sustainable food consumption (SFC) behaviour, social norms and ethical behaviour: “The under-consideration students” and “The negatively positioned students”. None of them has a high level of SFC, but the first and larger segment is more positively predisposed towards it.	Low
Makiniemi and Vainio, 2013, Finland	*n* = 350, (80), *M* = 24 SD 7.05	Climate-friendly food choices	Perceived moral intensity of climate change	Probable Seriousness of Consequences was by far the most important of the three moral intensity dimensions.	Low
Mäkiniemi and Vainio, 2014, Finland	*n* = 350, (80), *M* = 24 SD 7.05	Climate-friendly food choices	Perceived barriers	Wanting to eat the same as before, Disbelief in climate effects of food choices and lack of time had the greatest negative effect on climate-friendly food choices. Being male decreases the likelihood of choosing climate-friendly foods.	Low
Mohd Suki and Mohd Suki, 2015, Malaysia	*n* = 700, (55), NR Range 18–25	Green food consumption behaviour	Religion: Muslim vs non-Muslim (Hindus and Buddhists), specific needs, convenience, intention, promotion/diffusion, governmental efforts	Muslim consumers have lower scores on the evaluated factors, except for convenience factor correlation. Specific needs were the main contributing factor and the strongest predictor in discriminating between Muslim and non-Muslim consumers’ green food consumption.	Low
Schoolman, 2019, United States	*n* = 2,328, (52,9), NR	Ethical food consumption	Emotional experience of shopping	Purchasing “ethical” or “sustainable” foods is associated with experiencing shopping for food as enjoyable.	Moderate
Vecchio and Annunziata, 2013, Italy	*n* = 500, (58.6), NR	Sustainable food products purchase behaviour	Personality, attitudes, values, lifestyles, demographic and socio-economic characteristics.	Responsible food consumer cluster consisted of: urban citizens, live alone or with other students, medium-high household incomes, higher number of worker-students. Inattentive food consumer cluster consisted of: low degree of knowledge of the main sustainability issues, low-involvement attitude to virtuous lifestyle habits, do not think that their generation is adopting unsustainable consumption patterns, non-urban areas and families with medium household income. Potentially sustainable food consumer consisted of: least satisfied with the available information on sustainable food, majority of students that live in non-urban areas and are part of families with a medium household income.	Moderate

a n, sample size.

b Examined factors, in addition to demographics such as sex, age.

c NOS, New-Castle Ottawa Scale.

d Multiple countries: China, Czech Republic, United Kingdom, Iran, Ireland, South Korea, Macedonia, Norway, Serbia, Spain, and Sweden.

NR, no reported; M, mean, SD, standard deviation.

### Study Population and Measurement

There were four multi-country studies [26–29] and in total 30 represented countries. The top frequencies of study locations were 10 from the United States (US) [30–39], five from Italy [40–44], and three from Spain [45–47]. All the included papers were cross-sectional and were based on 38 unique samples. Ten articles addressed an umbrella concept (e.g., sustainable, green or climate-friendly food consumption) and measured several target behaviours [31, 37, 43, 48–54], while the rest reported a single outcome relevant for the analysis. Almost a third of the articles adopted a specific theoretical or conceptual framework for hypothesis formation and measurement. The most common was the Theory of Planned Behaviour (TPB) [28, 41, 54–56]. All the 40 studies identified evaluated personal factors while 11 (29%) also included social or situational factors. Being a woman was reported as a factor related to SFC in eight out of the twelve articles that reported significant gender-related differences. Three reported lower levels of food waste in men. The mean age of the study participants ranged from 18 to 29 years. On average, 60.7% were women and two studies were conducted with female students only.

### Behavioural Categories of Sustainable Food Consumption (SFC)

**FIGURE 2:**
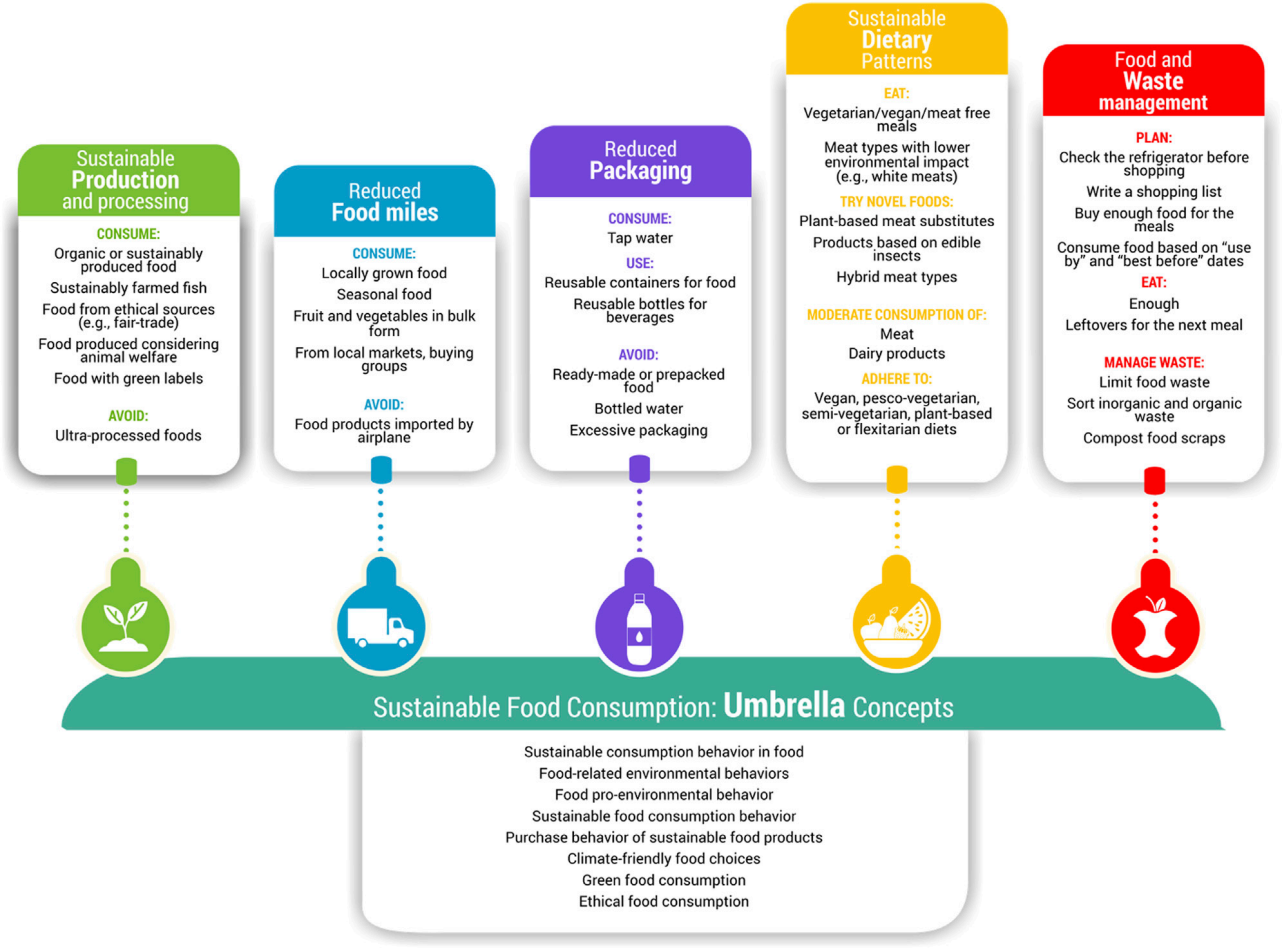
Categorization of Sustainable Food Consumption (SFC) behaviours, and behavioural outcomes extracted from the selected publications after data reduction. (What influences the sustainable food consumption behaviours of university students? A systematic review, several countries, 2021).

This section summarizes the findings about factors related to the observed sustainable, and unsustainable, food consumption behaviours of university students. The results are divided into proposed operational categories of Sustainable Food Consumption (SFC) behaviours. Figure 2 presents the proposed categories and summarizes the corresponding target behaviours extracted from articles. An exhaustive list of behavioural outcomes was extracted from the selected articles, and data reduction of similar behaviours was performed until reaching saturation.

The articles analysed related to a broad range of sustainable food consumption behaviours of university students, from “farm to dump,” reflecting food consumption choices based on a) how food is produced (e.g., organic), b) the environmental impact of food transport or “food miles” (e.g., consumption of local products), c) food packaging, d) specific foods choices or dietary patterns (e.g., plan-based diets, moderate meat consumption), and e) food waste. Most studies in our sample focused on dietary behaviours, followed by food waste. The frequencies of publications per category are presented in the PRISMA flowchart (See Figure 1). Outcomes related to air-transported foods avoidance, consumption of seasonal products, or those with low environmental impact (e.g., efficient water, land use, sustainable fisheries) were covered by articles that examined SFC behaviour as an umbrella concept. No studies about cultured meat were eligible for inclusion.

A higher proportion of students already consume organic food, with reports of frequent consumption ranging from 44% [57] to 89%, [58]; seasonal and local food products, reported as the top SFC behaviours by Kamenidou et al. [51] with mean scores of 5.46 and 5.10 out of 7, respectively; avoid some meats (47.4%) [26] or avoided plastic bottled water (34%) [47]. This contrasts with the relatively lower prevalence of self-declared “flexitarians” (15.4%) [33], “pescovegetarians” (11.6%) [33], “semi-vegetarians,” 6% [29] to 12.1% [33], vegetarians, ranging from 3.9% [26] to 25% [59], and vegans, ranging from 0.4% [26] to 1.8% [59].

### From the Farm: Sustainable Production

Six studies were focused on consumption of organic food (OF) [32, 34, 40, 57, 58, 60]. Three articles reported that knowledge and attitudes about OF had a positive relationship with the purchase and consumption of these foods (Correlation coefficients (r) between 0.24 and 0.28) [32, 34, 58]. Perceived safety, nutritional value and the perception that organic is fresher and has better taste, were also factors correlated with organic food consumption [58]. Positive associations were also found between the knowledge score of students and organic food consumption. McReynolds et al. [34] found that students with experience growing food had a higher frequency of OF purchase consumption (Chi^2^ *p* = 0.01) and organic fruit consumption (Chi^2^ *p* = 0.02) compared to students without such experience. Females had greater intention to buy organic food but there were no differences in consumption compared to males [34]. A perceived health risk reduction of OF consumption was associated with incremented frequency of OF consumption [40] and Green Perceived Value (GPV) constructs, especially emotional value, had a significant positive effect on purchase intentions, which in turn, had a positive effect on purchase behaviour moderated by food neophobia. In contrast, reported barriers to buying OF were higher price (35.9%), OF perceived as not attractive (20.5%), and distrust in OF being “better” or “non-chemical” (19.8%) [61].

### Reduced Food Miles

One study examined the association of local identity, brand valuation and the moderating effect of perceived availability on purchasing four local brands of tomato sauce, rice, mineral water, and a traditional local cake. A direct effect of local identity on effective purchase was only found for the local brand of mineral water, while there was a positive indirect effect of local identity through brand valuation for tomato sauce, rice, and cake brands. This indirect effect was further conditioned to the perceived availability of the tomato sauce and rice brands [45].

### Reduced Food Packaging

One article compared the frequency of tap water consumption with the frequency of bottled water consumption. Compared to university faculty and staff, students were the most frequent consumers of bottled water (43.9 and 39.3%, respectively). Agreement (one total disagree to five total agree) with the statement “it is safer to drink bottled water than tap water” varied among bottled water consumers (Kruskal-wallis *p* = 0.00), multiple comparison showed that differences arose from consumers of ≥6 bottles per week having a median of 3 range one to four compared to those consuming one to five bottles per week (Median 2 range 1–2) [47].

### The Fork: Sustainable Dietary Patterns

Fourteen articles assessed food-based behaviours. Eleven examined the adherence to full dietary patterns and three addressed more narrowly the consumption or substitution of meat and animal products.

Studies that examined factors associated with adherence to vegetarian diets [35, 38, 39, 59, 62, 63] found that a vegetarian diet pattern was associated with being female, non-smoker, lower proportion of daily caloric intake from fats, a lower-income, and use of vitamin-mineral supplements. Body mass index (BMI) and physical activity yielded mixed results [35, 38, 59]. Spencer et al. [38], found vegetarians had BMI ≤ 25 [38], Suleiman and colleagues [59] found that vegetarianism was associated with a normal BMI and being physically active among students in Jordan [59], while Olfert et al. [35], did not find significant differences in BMI and physical activity levels between vegetarian and non-vegetarian students in the USA. Surprisingly, Barros et al., in a model adjusted by sex, age, BMI, cohabitants and major, found that students who reported prejudicial alcohol had an 2.6% (95%CI 1.4;4.7) increased odds of adopting vegetarian diet [62], and Olfert et al [35] found higher stress levels among vegetarians.

Forestell and colleagues [64] examined food restraint, demographic, personality and lifestyle characteristics among vegetarian, pesco-vegetarian, semi-vegetarian and flexitarian compared to omnivores. Vegetarians and pesco-vegetarians were more open to new experiences, variety seeking, and had less food neophobia. Vegetarians and pesco-vegetarians did not differ from omnivores in their restraint level, while semi-vegetarians and flexitarians were more restrained than omnivores.

Two studies addressing plant-based diets took a more specific approach to understand factors for successful adherence in the USA, and the role of mindful eating on the adherence to healthful vs. unhealthful plant-based diets in Japan [36, 65]. Successful adherents to a plant-based diet, compared to those who tried without success, had higher levels of value, self-efficacy, planning/stimulus control and positive affect, while self-monitoring and self-criticism were negatively correlated. They were also seventeen times more likely to report “To manage or treat a medical condition,” almost seven times more likely to report “To align with my ethical beliefs,” and 94% less likely to report “To maintain and/or improve my health.”

Students who had higher scores for healthful plant-based diet (hPDI-J) also had higher total “health of the planet” and “awareness and appreciation for food” mindful eating sub-scores. Instead, “non-judgmental awareness” was correlated with a low intake of healthful plant-based foods. Smith et al [39] compared groups of students who had followed vegetarian and/or weight-loss diets and found that the vegetarian group could adhere to their diet for longer. The top reasons to drop the vegetarian diet were missing meat and concerns about nutrient intake [39]. A study in Albania found very low adherence to the EAT-Lancet reference diet and did not find any associations with the factors of interest (BMI, cost and eating out of home) [66]. Lower adherence to the Mediterranean Food Pyramid reference diet and higher meat consumption was found in a cluster of younger students, more females and living with parents [44].

Three articles examined factors concretely related to meat consumption, such as meat avoidance [26], beef consumption [27], and consumption of an insect-based product [41]. A study conducted in 11 countries across Europe and Asia found significant differences regarding the reasons to avoid meat among different groups of meat avoiders. The environment was the main reason for those who avoid some meats; health was the most important reason for most vegetarians, whereas vegans were most concerned about animal welfare-related reasons [26]. Beef consumption frequency was significantly correlated with being male in the USA, France, Brazil, but not in Argentina [27]. Intention and Perceived Behavioural Control (PBC) were the main predictors of tasting an insect-based food product. Students enrolled in social sciences were less likely to taste cricket flour than those in food and environmental sciences [41].

### To the Dump: Food and Waste Management

Two main behavioural outcomes were studied. Two studies measured self-reported food waste reduction behaviours (e.g., making a shopping list, using leftovers) [30, 42], while five observed the amount of food waste (plate waste/leftovers) [46, 55, 56, 67, 68], and one assessed both [28].

Three out of the eight articles dealing with food waste reported significant associations with gender. One study in Spain found a significant association between higher food waste and being female [46], while two other studies found that females wasted slightly more meat [67] and staple [56]. Higher-income/living expenses were associated with higher food waste in two studies [42, 56]. Composting [30], “addiction to sales,” [28] concerns about food safety, and lack of knowledge about food waste (the belief that only 10% of the food purchased gets thrown away, not knowing that waste is a more serious problem than packaging) [42] were negatively associated with food waste reduction behaviours.

Three papers that examined TPB constructs had mixed results. Alattar and colleagues found that attitudes and intent were the strongest predictors of food waste diversion behaviours among university students in the USA [30]. In contrast, Mondejar-Jimenez et al. [28] found among their student sample in Italy and Spain that the strongest predictor of (correct) behaviour towards food waste were subjective norms followed by PBC. Wu et al. [56] found that more food was wasted in association with low PBC in China, while subjective norms and attitudes had no significant association.

Lorenz et al. [55] explored personal, social and environmental (situational) determinants associated with leftover behaviour, revealing interactions between personal and environmental factors. While time pressure was not a direct environmental determinant of leftovers, being female becomes a significant determinant for this behaviour among students under time pressure. There was a significant relationship between perceptions of food (portions size and palatability) and food leftovers. No significant association was found between the presence of others and food leftovers. In a later study, the same authors, also in Germany [68], broke down attitudes into more specific subsets of beliefs (self-interest, pro-environmental, resource efficiency), finding interactions between situational variables and self-interested beliefs.

### Umbrella Concepts

Ten of the included articles addressed SFC, integrating different behaviours measured by an index, composite measure or score (See Supplementary Material). Students “in a relationship” had higher SFC levels than “single” (Mean Difference = −0.16, *p* < 0.05) [49]. High levels of “environmental awareness and action” (engaging in other behaviours such as energy-efficient cooking, avoid plastic waste, and sorting inorganic and organic food) (*β* = 0.46, *p* < 0.05) [49], intention (*β* = 0.74, *p* < 0.001), perceived seriousness of consequences of climate change (*β* = 0.10, *p* < 0.05) [52], biospheric value orientation (BVO) (*β* = 0.28, *p* < 0.001), and environmental beliefs (*β* = 0.24, *p* < 0.001) [31] were associated with SFC behaviours. Attitudes (*β* = 0.28, *p* < 0.001) and knowledge on the environmental impact of food consumption (*β* = 0.14, *p* < 0.01) also were associated with SFC [50]. Being male was associated with lower SFC behaviour in two samples of students in the United States (*β* = −0.11, *p* < 0.05) [31] and Finland (*β* = -0.13, *p* < 0.01) [52]. There were no significant differences in green food consumption between Muslim and non-Muslim students in Malaysia, despite significant differences in personal needs, environmental values and perceptions about government efforts related to green food [54]. No other demographic characteristics were significant predictors of SFC.

Two groups of researchers took a factor analysis approach to identify student segments. Kamenidou et al. [51] identified two segments based on SFC behaviour, social norms, and ethical behaviour: “The under-consideration students” and “The negatively positioned students”. None of the segments show higher SFC levels, but the first and larger segments were positively predisposed towards it. Frequent SFC behaviours were limited to seasonal and local food consumption. Vecchio and Annunziata [43] identified three clusters: “responsible food consumer” (urban citizens, live alone or with other students, medium-high household incomes, higher amount of worker-students), “inattentive food consumer” (low degree of knowledge of sustainability issues, low-involvement attitude to virtuous lifestyle habits, and medium household income), and “potentially sustainable food consumer” (least satisfied with the available information on sustainable food, majority of students that live in non-urban areas, medium household income). Under the label of “ethical food consumption,” Schoolman [37] measured purchase frequency of products that can fall into SFC (locally grown/processed food, organic food, fair trade food, food from humanely treated animals, and fish from sustainable fisheries). This study found that, for each additional point on the ethical food consumption index, students were 51.1% more likely, to declare they enjoyed shopping food daily.

### Quality Assessment

Scores for the included papers ranged from 3 to 8, out of 10 possible points, with a median of 5 points; 75% of the articles were classified as low quality and 25% rated moderate. Out of the 17 studies that implemented regression methods, seven adjusted or stratified by sex or age. Thirty-nine studies did not report the response rates, and 26 did not justify the sample size. The quality assessment scores of selected studies are listed in the supplementary material.

## Discussion

Based on data from 40 included publications, we found that literature evaluating the related factors associated with SFC behaviours has focused mainly on personal factors, such as intention, knowledge, attitudes, lifestyle, values and beliefs, and there is scarce evidence on social and environmental (situational) factors.

A higher proportion of students already consume organic, seasonal and local food, fewer avoid some meats, and there is a relatively low prevalence of self-declared “flexitarians,” “pescovegetarians,” “semi-vegetarians,” vegetarians, and vegans. This shows a higher reported adoption of SFC behaviours with lower planetary health potential: while the sustainability of organic food can be limited [69] and organic production is only one of many forms of sustainable agriculture, the livestock sector contributes to an estimated 14.5% of the total human-induced GHG emissions [70].

### Underlying Factors and Characteristics of Sustainable Food Consumers

Except for food waste [46, 56], being a woman was reported as a factor related to SFC [31, 59], but situational factors moderated this association (e.g., time pressure) [55]. Factors such as knowledge and attitudes yielded mixed results. Concern about food safety was positively associated with organic food consumption [58] but negatively associated with food waste prevention [42]. Composting was associated with higher food waste [30].

Concerning lifestyles, sustainable consumers tended to have healthier lifestyles, better dietary habits [35, 38, 59], and enjoy food shopping more [37] than less sustainable consumers. Similarly, students were able to adhere to vegetarian diets for longer than to weight-loss diets [39]. However, weight control and food restraint associated with SFC require further analyses as they can incur health risks, and the healthfulness and sustainability of eating behaviours are often dose-dependent. Two studies found that vegetarians had higher levels of stress [35] and prejudicial alcohol consumption [62], in samples of university students in the United States, and Brazil. These adverse associations deserve further examination. Similar attention is needed about the consumption of plant-based meat substitutes, included as outcome in one of the selected studies [51] as they have the benefits of vegetable consumption but can be highly processed.

Significant associations were found between knowledge and outcomes for organic food consumption [34] and making a shopping list (food waste prevention) [42]. Conversely, the lack of knowledge on the environmental impact of food was associated with less sustainable food consumption [50]. In particular, participants underestimated the environmental impact of meat consumption [50] and food waste [42], and overestimated the impact of other behaviours such as food packaging [42]. This low awareness about the environmental impact of food choices is aligned with previous findings [71]. Other studies did not find significant associations between knowledge and organic food consumption [32, 58], suggesting the need for further examination to disentangle the mechanisms involved in knowledge as a predictor of behaviour.

Behavioural outcomes, such as meat reduction and avoidance, were associated with different factors depending on the reported motivations for eating behaviour (health, environment, animal rights). This is compatible with literature on factors linked to different eating motives [72, 73].

### Operational Categorization of Sustainable Food Consumption Behaviours

There is still a lack of operational, standardized behavioural definitions of sustainable food consumption from the consumer perspective [12, 74]. Dietary behaviours, such as adherence to vegetarian or flexitarian diets, followed by food waste, were the most studied behavioural categories. While the categories are not meant to be exhaustive, they cover a diverse variety of behaviours.

The lack of behavioural measures was a common reason for exclusion of otherwise eligible studies. Measuring behaviour can be challenging when studying food intake, especially when these behaviours are uncommon (e.g., cultured-meat, edible insects, meat-mushroom blends). Menozzi et al. [41] on cricket flour presents a sound methodological solution to overcome this problem. Behavioural data collected in virtual reality is also a promising alternative as data can be comparable to real-life consumption data [75].

### Strengths and Limitations

To the author’s knowledge, this is the first literature review integrating a broad range of sustainable food consumption behaviours for a specific population. This comes with the challenge of synthesizing a diversity of outcomes measured in different ways, as studies included were highly heterogeneous, which was a barrier for meta-analysis. However, examining SFC as an umbrella concept, allowed identifying possibly conflicting interactions between different behaviours and factors that would not be possible when reviewing articles for a single target behaviour. We followed a strict definition of behavioural outcomes, excluding studies that did not include self-reported or observed behavioural measures. This was essential to answer the main research question but excluded many otherwise eligible studies that measured acceptance, willingness, attitudes, or behavioural intention, possibly affecting the geographic coverage and variety of target behaviours captured by the review.

The selected studies covered three continents and 30 countries in all income economies levels. Relevant studies that exceeded the scope of this review, e.g. qualitative, case studies, focused on awareness, etc., have been conducted in other countries [18, 76–85]. Since most of the selected studies rely on convenience sampling, generalizations about the country population are not possible. Yet, relative homogeneity of the population supports conclusions about young adults and university students with caution.

The examination of social and situational factors is rather neglected in the selected studies at hand. This may be due to higher interest in personal factors, the fact that some social or environmental/situational factors are classified as personal, but it may also be due to the observational nature of the study designs covered in this review. Other reviews conducted on experimental study designs on meat consumption, for example, yielded more balanced proportions of personal and environmental/situational determinants [86–88].

### Conclusion

Our findings support previous evidence about the health and environmental co-benefits of sustainable food consumption [7–10], from the consumer behaviour perspective. Healthy lifestyles of sustainable food consumers suggest possible synergies between environmental and health motivations of food choice and longer-term adherence to healthy diets. Future research areas can examine the effects of communication framings that emphasize the individual health or pro-social environmental benefits of SFC in different populations. There is also a need to further examine the behavioural aspects related to the co-benefits and also the management of risks associated to SFC, at the lifestyle and health outcomes level. Social norms [89, 90], including related variables as eating with others, and situational factors such as time pressure, portion size, palatability [55], availability of sustainable alternatives, food repositioning or labelling [86] deserve further examination. The moderation effects of social and environmental factors on personal factors related to sustainable food consumption reveal opportunities to design choice architecture interventions. Future research could evaluate the interaction between possibly conflicting predictors of different SFC behaviours, the disentangling mechanisms behind attitudes and knowledge as a predictor of behaviour, and the factors to adopt SFC in male consumers.

Practical implications include: for universities, the need for monitoring the effects of their food environments, and situational factors, on the food choices of students; for key actors in the production side of the food chain, the almost absent sustainable produced food consumption alternatives, beyond organic food, show the need for more transparency about other aspects of production sustainability that are increasingly relevant for young consumers; and for food policy actors this work adds to the growing body evidence about diverse SFC behaviours that can be promoted to advance health and sustainability targets. The proposed categorization of behaviours is not meant to be exhaustive but contributes to the behavioural operationalization of sustainable food consumption including but not limited to sustainable diets.

From a planetary health perspective, the sustainability of food consumption becomes a pressing public health issue, as it is recognized that adverse effects on population health result from unsustainable and unhealthy food consumption. This urgency is consistent with an evolving view of sustainable development that acknowledges that healthy economies and societies depend on the life-sustaining capabilities of the ecological system [91].
