# Supplementary material for: What Influences the Sustainable Food Consumption Behaviours of University Students? A Systematic Review
Source: Int J Public Health. 2021 Sep 7;66:1604149. doi: 10.3389/ijph.2021.1604149 (PMC8454891; doi:10.3389/ijph.2021.1604149)
Supplement: Supplementary file 1 [file DataSheet1.zip › Supplement_Aguirre-2021_SFC-review_18082021.pdf]

What influences the sustainable food consumption behaviours of university students? A systematic review

Supplementary Material

1. Search Strategy per database 2
2. Journals screened by hand 5
3. Data extraction items 5
4. Quality assessment of cross-sectional studies by using the Newcastle Ottawa Scale . 6
5. Inventory of factors, behaviors and summary of frequencies, per category 8

Supplementary Material

1. Search Strategy per database

Medline Ovid (27.01.2021, 1590 records)

Ovid MEDLINE(R) and Epub Ahead of Print, In-Process& Other Non-Indexed Citations, Daily and Versions(R) <1946 to January 26, 2021>

1exp Students/ or Young Adult/ or Universities/1020579

2(student* or universit* or undergraduate* or postgraduate* or college* or "tertiary education" or campus or young adult* or young consumer* or young person* or young people or youth or "generation X" or "generation Z" or millennials).ab,ti.887522

31 or 21698739

4Feeding Behavior/ or Food Preferences/ or Choice Behavior/ or Consumer Behavior/144991

5attitude/ or attitude to health/ or health knowledge, attitudes, practice/238579

6(behav* or choice* or consum* or purchas* or buy or shop* or intake or habits or habit or habitual or pattern* or attitud* or aware* or perception* or perceive or prefer* or select* or knowledge*).ti,ab.6301909

74 or 5 or 66425422

8((sustainab* or farm-to-fork or green or climate-friendl* or ecological* or environmentally friendl* or environmentally conscious* or eco-friendl* or fair-trade or mindful* or organic or plant-based or vegetarian* or vegan* or flexitarian*) adj6 (diet* or food* or nutri* or eating* or consum* or meal or meals)).ab,ti.23373

9((food or plate) adj3 (wast* or leftover*)).ab,ti.3747

10(food-print or foodprint).ti,ab.6

11((local or locally or farm-fresh or season*) adj2 (food or foods or vegetable* or veggies or fruit*)).ti,ab.2892

12((avoid* or renounc* or give up or reduc* or curtail* or moderate or less or substitut* or plant-based) adj6 (meat or flesh food*)).ab,ti.3175

138 or 9 or 10 or 11 or 1232169

143 and 7 and 131873

1514 not (exp animals/ not humans/) not (letter or news or comment or editorial or congress or published erratum or guideline or review).pt. not ((exp infant/ or exp child/ or adolescent/) not (young adult/ or adult/))1590

Embase Ovid (27.01.2021, 1096 records)

Embase <1974 to 2021 January 26>

1exp student/ or young adult/ or university/722953

2(student* or universit* or undergraduate* or postgraduate* or college* or "tertiary education" or campus or young adult* or young consumer* or young person* or young people or youth or "generation X" or "generation Z" or millennials).ab,ti.1398697

31 or 21774059

4feeding behavior/ or food preference/ or eating habit/ or decision making/ or consumer attitude/344077

5attitude/ or attitude to health/181276

6(behav* or choice* or consum* or purchas* or buy or shop* or intake or habits or habit or habitual or pattern* or attitud* or aware* or perception* or perceive or prefer* or select* or knowledge*).ti,ab.7794097

74 or 5 or 68010271

8((sustainab* or farm-to-fork or green or climate-friendl* or ecological* or environmentally friendl* or environmentally conscious* or eco-friendl* or fair-trade or mindful* or organic or plant-based or vegetarian* or vegan* or flexitarian*) adj6 (diet* or food* or nutri* or eating* or consum* or meal or meals)).ab,ti.27351

9((food or plate) adj3 (wast* or leftover*)).ab,ti.4427

10(food-print or foodprint).ti,ab.7

11((local or locally or farm-fresh or season*) adj2 (food or foods or vegetable* or veggies or fruit*)).ti,ab.3524

12((avoid* or renounc* or give up or reduc* or curtail* or moderate or less or substitut* or plant-based) adj6 (meat or flesh food*)).ab,ti.3426

138 or 9 or 10 or 11 or 1237518

143 and 7 and 131921

1514 not (exp animal/ not human/) not (letter or note or editorial or conference or erratum or review).pt. not ((exp child/ or exp adolescent/) not (young adult/ or adult/))1096

PsycInfo Ovid (27.01.2021, 478 records)

APA PsycInfo <1806 to January Week 3 2021>

1exp students/ or colleges/278570

2(student* or universit* or undergraduate* or postgraduate* or college* or "tertiary education" or campus or young adult* or young consumer* or young person* or young people or youth or "generation X" or "generation Z" or millennials).ab,ti.812063
31 or 2873990
4Eating Behavior/ or Food Preferences/ or Eating Attitudes/ or Choice Behavior/ or exp Consumer Behavior/66786
5Attitudes/ or Health Attitudes/ or Health Knowledge/ or Health Literacy/47567
6(behav* or choice* or consum* or purchas* or buy or shop* or intake or habits or habit or habitual or pattern* or attitud* or aware* or perception* or perceive or prefer* or select* or knowledge*).ti,ab.2244805
74 or 5 or 62260733
8((sustainab* or farm-to-fork or green or climate-friendl* or ecological* or environmentally friendl* or environmentally conscious* or eco-friendl* or fair-trade or mindful* or organic or plant-based or vegetarian* or vegan* or flexitarian*) adj6 (diet* or food* or nutri* or eating* or consum* or meal or meals)).ab,ti.3630
9((food or plate) adj3 (wast* or leftover*)).ab,ti.266
10(food-print or foodprint).ti,ab.3
11((local or locally or farm-fresh or season*) adj2 (food or foods or vegetable* or veggies or fruit*)).ti,ab.592
12((avoid* or renounc* or give up or reduc* or curtail* or moderate or less or substitut* or plant-based) adj6 (meat or flesh food*)).ab,ti.303
138 or 9 or 10 or 11 or 124575
143 and 7 and 13574
1514 not (letter or comment or editorial or abstract or erratum or review).dt.571
16*15 not (("140" or "160" or "180" or "200") not "300").ag.478
**140 Infancy, 160 Preschool Age, 180 School Age, 200 Adolescence, 300 Adulthood*

Web of Science Core Collection (27.01.2021, 2137 records)

12 #11 AND dt=(article)2137
11 #10 AND #4 AND #3 2503
10 #9 OR #8 OR #7 OR #6 OR #591366
9 TS=((avoid* or renounc* or "give up" or reduc* or curtail* or moderate or "less" or substitut* or "plant based") NEAR/5 ("meat" or "flesh food*"))5177
8 TS=((local or locally or "farm fresh" or season*) NEAR/1 (food or foods or vegetable* or veggies or fruit*)) 9268
7 TS=("food print" or foodprint)23
6 TS=((food or plate) NEAR/2 (wast* or leftover*)) 13194
5 TS=((sustainab* or "farm-to-fork" or "green" or "climate friendl*" or ecological* or "environmentally friendl*" or "environmentally conscious*" or "eco friendl*" or "fair trade" or mindful* or organic or "plant-based" or vegetarian* or vegan* or flexitarian*) NEAR/5 (diet* or food* or nutri* or eating* or consum* or "meal" or "meals"))66967
4TS=(behav* or choice* or consum* or purchas* or "buy" or shop* or intake or habits or habit or habitual or pattern* or attitud* or aware* or perception* or perceive or prefer* or "select" or "selected" or "selection" or knowledge*)12828682
3 #2 OR #11345158
2 TS=(universit* NEAR/3 (cafe* or canteen* or lunchroom or "dining hall" or restaurant*))496
1 TS=(student* or undergraduate* or postgraduate* or "university graduat*" or graduate college* or "tertiary education" or campus or young adult* or young consumer* or young person* or young people or youth or "generation X" or "generation Z" or millennials) 1344868

Scopus (27.01.2021, 1989 records)

(((TITLE-ABS-KEY (student* OR undergraduate* OR postgraduate* OR "university graduat*" OR college* OR campus OR "young adult*")) OR (TITLE-ABS-KEY (universit* W/2 (cafe* OR canteen* OR lunchroom OR "dining hall" OR restaurant*)))) AND (TITLE-ABS-KEY (behav* OR choice* OR consum* OR habits OR pattern* OR attitud* OR aware* OR perception* OR perceive OR prefer* OR "selection" OR knowledge*))) AND ((TITLE-ABS-KEY (((sustainab* OR "farm-to-fork" OR "green" OR "climate friendl*" OR ecological* OR "environmentally friendl*" OR "environmentally conscious*" OR "eco friendl*" OR "fair trade" OR mindful* OR organic OR "plant-based" OR vegetarian* OR vegan* OR flexitarian*) W/3 (diet* OR food* OR eating* OR consum* OR "meal" OR "meals")))) OR (TITLE-ABS-KEY (((food OR plate) W/2 (wast* OR leftover*)))) OR (TITLE-ABS-KEY ("food print" OR foodprint)) OR (TITLE-ABS-KEY (((local OR locally OR "farm fresh" OR season*) W/1 (food OR foods OR vegetable* OR veggies OR fruit*)))) OR (TITLE-ABS-KEY (((avoid* OR renounc* OR "give up" OR reduc* OR curtail* OR moderate OR "less" OR substitut* OR "plant based") W/3 ("meat"))))) AND (LIMIT-TO (DOCTYPE , "ar")) AND (EXCLUDE (EXACTKEYWORD , "Child") OR EXCLUDE (EXACTKEYWORD , "Aged,

80 And Over") OR EXCLUDE (EXACTKEYWORD , "Very Elderly") OR EXCLUDE (EXACTKEYWORD , "Preschool Child") OR EXCLUDE (EXACTKEYWORD , "Child, Preschool") OR EXCLUDE (EXACTKEYWORD , "School Child"))
**LILACS** (via VHL Regional Portal) **(27.01.2021, 105 records)**
(student* OR "young adult" OR "young adults" OR "young consumers" OR "young persons" OR "young people" OR universit* OR undergraduate* OR postgraduate* OR college* OR campus OR youth) AND ("sustainable food" OR "sustainable diet" OR "food sustainability" OR "sustainable consumption" OR "food print" OR foodprint OR "sustainable eating" OR "farm-to-fork" OR "green food" OR "climate-friendly food" OR "climate-friendly meals" OR "climate-friendly eating" OR "environmentally friendly" OR "environmentally conscious" OR "environmental consciousness" OR "ecological food consumption" OR "ecological food choice" OR "eco-friendly" OR "eco-friendliness" OR "fair-trade" OR "plant-based" OR vegetarian* OR vegan* OR flexitarian* OR "meat avoidance" OR "avoiding meat" OR "avoid meat" OR "less meat" OR "moderate meat consumption" OR "reduced meat consumption" OR "meat substitution" OR "meat substitute" OR "organic food" OR "food waste" OR "local food" OR "local foods" OR "locally-grown food" OR "seasonal food" OR "seasonal foods" OR "seasonal fruit" OR "seasonal fruits" OR "seasonal vegetables" OR "farm-fresh") AND (db:("LILACS"))<br>*Remarks:*<br>*- Concept 2 (attitude/behaviour) not included, since the other two concepts generate only few results.*<br>*- No limitation for human studies only or age groups: not possible in LILACS*<br>*- No truncation possible in phrases*
**Google Scholar (27.01.2021, first 200 out of 14800 records)**
"university|college students"|"young adults|people" attitude|behavior|consumption|choice|purchase|habit|preference|selection "sustainable food|diet|nutrition|meals"|"organic|eco-friendly|environmentally-conscious|local|seasonal food|vegetables|fruits"

2. Journals screened by hand

a. Appetite
c. Journal of Consumer Behavior
d. Sustainable Production and Consumption
e. Public Health Nutrition
f. International Journal of Environmental Research and Public Health
g. International Journal of Public Health
h. Lancet Planetary Health
i. International Journal of Sustainability in Higher Education

3. Data extraction items

The data extraction form comprised the following topics:

- Identification: 1) Reviewer's name; 2) DOI; 3) Author/s, year; 4) study location (Country where the study was conducted

- Sample characteristics: 1) Sample size; 2) Percentage of women; 3) Age of the sample; 4) Composition rural/urban; 5) Religious affiliation; 6) education level (e.g., undergraduate, graduate, PhD); 7) sample characteristics; 8) income level; 9) ethnicity; 10)

- Study characteristics: 1) study design; 2) behavioral outcome measured; 3) specific behavior/s addressed in the outcome (e.g., choice, intake, purchase); 4) description of factors/exposures measured in the study; 5) statistical test implemented for the evaluation of association; 6) theoretical approach/conceptual framework; 7) type of statistical analysis conducted; 10)

- Results: 1) Type of quantitative association measures reported (e.g odds ratio, beta estimates); 2) Confounding factors are controlled, list confounders; 3) human health effect reported; 4) environmental outcomes reported; 5) Conclusions

4. Quality assessment of cross-sectional studies by using the Newcastle Ottawa Scale

Author/s, year.	Selection				Comparability		Outcome		Score/Classification
	Representativeness of the sample	Sample size	Non-respondents	Ascertainment of the exposure (factors)	Control for the most important factor (sex, age)	Control for additional factors	Assessment of the (behavioral) outcome	Statistical test:	
**Akbar et al., 2019**	1	1	0	1	1		1	1	6 / Moderate
**Alattar et al., 2020**	1	1	1	1	1		1	1	5 / Low
**Al-Domi H, 2011**	1	0	0	1	0		2	1	5 / Low
**Anh et al., 2019**	0	0	0	1	0		1	1	3 / Low
**Anh et al., 2020**	1	0	0	2	1		1	1	5 / Low
**Barros et al., 2020**	1	1	0	2	1	1	1	1	6 / Moderate
**Campbell-Arvai, 2015**	1	0	0	2	1		1	1	5 / Low
**Dahm et al., 2009**	1	0	0	1	0		1	1	4 / Low
**Díez et al, 2018**	1	1	1	2	0		1	1	7 / Moderate
**Dopelt et al., 2019**	1	0	0	1	1		1	1	5 / Low
**Fernandez-Ferrin et al., 2017**	0	0	0	1	1		1	1	4 / Low
**Forestell et al., 2012**	0	0	0	2	1		1	1	5 / Low
**Forleo et al., 2017**	1	0	0	1	0		1	1	4 / Low
**Giampietri et al., 2020**	0	1	0	1	1		1	1	5 / Low
**Hamilton and Hekmat, 2018**	1	1	0	0	1		1	0	4 / Low
**Izmirli and Phillips, 2011**	1	0	0	1	0		1	1	4 / Low
**Kamenidou et al., 2019**	0	1	0	1	1		1	0	4 / Low
**Kawasaki et al., 2021**	1	1	1	2	1		1	1	8 / Good
**Llanaj and Hanley-Cook, 2020**	0	1	0	1	1		1	1	6 / Moderate
**Lorenz et al., 2017**	0	0	0	2	1		2	1	5 / Low
**Lorenz et al., 2018**	0	0	1	1	1		2	1	6 / Moderate
**Mäkiniemi and Vainio, 2013**	0	0	0	2	1		1	1	4 / Low
**Mäkiniemi and Vainio, 2014**	1	0	1	1	0		1	1	5 / Low
**McReynolds et a., 2017**	1	1	0	2	1		1	0	5 / Low
**Menozzi et al 2017**	1	0	0	1	0		2	1	5 / Low
**Mohd Suki and Mohd Suki, 2015**	0	0	0	1	0		1	1	3 / Low

Author/s, year.	Selection				Comparability		Outcome		Score/Classification
	**Representativeness of the sample**	**Sample size**	**Non-respondents**	**Ascertainment of the exposure (factors)**	**Control for the most important factor (sex, age)**	**Control for additional factors**	**Assessment of the (behavioral) outcome**	**Statistical test:**	
**Mondejar-Jimenez et al., 2017**	1	0	0	2	1		1	1	5 / Low
**Morata Verdugo et al., 2020**	0	0	0	2	0		2	0	4 / Low
**Olfert et al., 2020**	0	1	0	2	1	1	1	0	5 / Low
**Pocol et al., 2020**	1	0	0	1	0		1	1	4 / Low
**Principato et al, 2015**	1	0	0	1	0		1	1	4 / Low
**Ruby et al. 2016**	0	0	0	1	1		1	1	4 / Low
**Schoolman. 2019**	1	1	0	1	1	1	1	1	7 / Moderate
**Smith et al., 2000**	0	0	0	1	0		1	1	3 / Low
**Spencer et al., 2007**	1	0	1	1	0		1	1	5 / Low
**Suleiman et al., 2009**	1	1	1	2	0		1	1	7 / Moderate
**Vecchio R and Annunziata A, 2013**	1	1	1	2	0		1	1	7 / Moderate
**Vizcaino et al., 2020**	1	0	1	2	1		1	1	7 / Moderate
**Wu, et al. 2019**	1	0	0	2	1	1	2	1	7 / Moderate
**Zámková and Prokop, 2013**	0	0	0	1	0		1	1	3 / Low

Wells GA SB, O'Connell D, Peterson J, Welch V, Losos M, Tugwell P. The Newcastle-Ottawa Scale(NOS) for assessing the quality of nonrandomized studies in meta-analyses. Available: http://www.ohri.ca/programs/clinical_epidemiology/oxford.htm. Accessed 19 February 2021

5. Inventory of factors, behaviors and summary of frequencies, per category

	Underlying factors and characteristics studied	Sustainable food consumption behaviors	Key Frequencies
**Choices on food Production and processing**	Green Perceived Value, via purchase intention [1]<br><br>Food neophobia. [1]<br><br>Knowledge and attitudes towards organic food [2] [3] [4]<br><br>Attitudes and behaviors regarding other eco-friendly practices [2]<br><br>Individual risk attitude [5]	Consume:<br>Organic food products [4] [6] [1–5,7] [8] [9] [10][11] [12]<br>Sustainably farmed fish [11] [9]<br>Food from ethical producers (fair-trade, respects workers' rights) [11] [12]<br>Food from humanely treated animals [12] [11]<br>Food with green labels [13]<br><br>Avoid:<br>Processed foods (reverse-scored) [6]	Highest: Hamilton and Hekmat, 2018 (Canada) report 89.1 % consume OF "sometimes" or "often".<br><br>Lowest: Zámková and Prokop, 2013 (Czech Republic) report 44% buy OF "sometimes" or "regularly".
**Choices based on Food miles**	Local identity, brand valuation, and moderating effect of perceived availability. [14]	Consume:<br>Locally grown food [10] [6] [8] [9] [9] [12] [15] [11][14]<br>Seasonal food [8] [9] [15] [12]<br>Fruit and vegetables in bulk form [9] [12]<br>From local markets, buying groups [12]<br><br>Avoid:<br>Food products imported by airplane [9] [15]	Eating local and seasonal foods were the most reported behaviors in Kamenidou and Dopelt, and the second most frequent after food waste avoidance in Makiniemi and Vainio, 2013<br><br>Avoiding air-transported products was among the least reported behaviors in Kamenidou, 2019 and Makiniemi and Vainio, 2013.
**Choices on food Packaging**	Perceptions about bottled and tap water [16]	Consume:<br>Tap water [16]<br><br>Use:<br>reusable containers for leftovers [10]<br>reusable bottles for beverages [6] [16]<br><br>Avoid:<br>Convenience/ready-made prepacked food [10] [9]<br>Bottled water consumption [16]<br>Excessive packaging [9]	Diez focused on packaging only, while Anh, Campbell, and Kamenidou included food packaging behaviors as part of a broader SFC composite measure.<br><br>The least reported behaviors in Anh, 2019 (Vietnam). "I avoid eating convenience food because of plastic waste" (2.56), and "I use containers instead of plastic wraps/ bags" 2.76 (Max. score: 5)<br><br>Most reported behaviors in Campbell (US). 43.1 % reporting that "Recycle glass, plastic and paper (food boxes, containers and bottles)", and " 33.4 %

			"Use reusable containers for leftovers and reusable bottles for beverages" very often.<br><br>"Avoid food products with excessive packaging" with a mean score of 4.19, out of 7, was interpreted as students are "not currently doing this but are willing to do it in the future" (Kamenidou)
**Dietary Patterns**	Lifestyle characteristics (e.g. drinking alcohol, smoking) [17] [18]<br><br>Food restraint, personality inventory, food neophobia, general neophobia, food choice, sensory appeal, price, familiarity, mood, ethical concern, eating attitudes. [18]<br><br>Attitudes towards animals, and perceived importance of world issues. [19]<br><br>Mindful eating [20]<br><br>Anthropometric measurements, dietary intake, dietary cost and eating out of home [21]<br><br>Attitudes, subjective norms, perceived behavioral control, intention [22]<br><br>Perceptions of campus environment, waist circumference, hip circumference, fruit and vegetable intake, fat intake, perceived stress, eating ettitudes. [23]<br><br>Attitudes toward beef, and toward vegetarians. [24]<br><br>Lifestyle characteristics. [25]<br><br>Self-regulatory system, variety of motivations [26]<br><br>Health-related outcomes[27]	Moderate/reduce consumption of:<br>Eat less meat [6] [9]<br>Eat meat types with lower environmental impact (e.g. reduce red meat consumption) [9] [24]<br>Dairy products [15]<br><br>Eat:<br>Vegetarian meals [6]<br>Vegan meals [6]<br>Eat plant-based meat substitutes [9]<br>Eat hybrid meat types [9]<br><br>Adhere to:<br>vegetarian, pesco-vegetarian, semi-vegetarian, plant-based, flexitarian, Mediterranean or EAT-Lancet diets [17–21,23,25–30]<br><br>Being willing to try novel foods:<br>Products based on edible insects (e.g. cricket flour) [22] [9]	Omnivorous 93,6, Strict vegetarian 0,7, Ovolactovegetarian 5,4, Lactovegetarian 0,1, Ovovegetarian 0,2 (Barros et al., 2019)<br><br>Vegetarians 22.91, pescovegetarians 11.66, semi-vegetarians 12.08, flexitarians 15.41, omnivores 37.91. (Forestell et al., 2012)<br><br>No meats avoided 48.3, Some meats avoided 47.4, Vegetarian 3.9, Vegan 0.4" (Izmirli and Phillips, 2011)<br><br>Beef consumption. Mean (times/month): Argentina 19.36, Brazil 22.61, France 11.30, US 8.54 (Ruby, et al)<br><br>45% had tried a vegetarian diet at some point in their lives (Smith)<br><br>Some authors measured prevalence of vegetarian diet as self-identification as vegetarian, resulting in 5.7% of the sample in the US (Olfert), 7.2% of a sample of medical students in the US (Spencer) and 23.9% of a sample of students in Jordan (Suleiman).<br><br>Vizcaino et al compared the adherents group (n 91; 6.1 %), students who self-identified as vegan, vegetarian or pescatarian for 12 months or longer, with the Non-adherents group (n 178; 11·9 %), of those who described themselves as currently trying to adhere to a plant-based diet but were not always successful.

	Reasons for discontinuing vegetarian vs. weight loss diets [28]		23% of the sample accepted to participate in the tasting experience and actually tasted a chocolate chip cookie containing 10% of cricket flour (Menozzi)
**Food and Waste management**	Demographic and socioeconomic characteristics [31]<br><br>Food management skills, food waste atttitutes/emotions, perception of cost, food waste knowledge, general sustainability beliefs, perception of personal impact [32]<br><br>Personal, social and environmental determinants. Personal (Attitudes, PBC, subjective norms, Intention, Personal Norms), social (Presence of other persons during lunch) and environmental/situational (palatability of food, food portion sizes, and time pressure) [33]<br><br>Concern about food waste, moral attitude, subjective norms, perceived behavioral control, marketing/sale addiction, intention [34]<br><br>Eating habits and level of physical activity [35]<br><br>Knowledge, attitudes (level of cocern), demographic and socioeconomic characteristics [36]<br><br>Attitudes, perceived behavioral control, subjective norms, personal attributes (e.g. major, income level), perceived university canteens characteristics (e.g. food is not tasty, too much food	Plan<br>I check the refrigerator before shopping [32]<br>I regularly plan my purchases by writing a shopping list [34]<br>I always make a list of what I need before shopping [36]<br>I don't make lists or plan meals before shopping (reverse) [32]<br>Buy enough food for the meals to avoid waste [10]<br><br>Consume<br>I consume food sparingly and effectively [10]<br>I reuse leftovers for the next meal [10] I eat leftovers [32] I regularly use leftovers in the following days [34]<br><br>Manage waste<br>I try to limit food waste [15]<br>I am aware of the differences between "use by" and "best before" dates [34]<br>I sort the inorganic or organic waste before throwing into the trash [10]<br>Throw food out (reverse-scored) [6]<br>Compost food scraps [6]	In Lorenz, 2017 (Germany) 75% hardly had leftovers, 15% moderate leftovers and 8% considerable leftovers.<br><br>Al-Domi estimated 13% plate waste (weight) and Morata-Verdugo estimated 14.5% of plate waste (visual estimation) in their respective samples in Jordan and Spain.<br><br>In Allattar an average of 18% of the food bought was reported as wasted. 59% of students in Italy, and 63% of students in Spain reported to waste 15% of their food. Mondejar-Jimenez<br><br>Other authors measured self-report of behaviors that prevent or reduce food waste. In Allatar et al, 82.4% report "I eat leftovers", and 77% report "I check the refrigerator before shopping". In Principato, the following behaviors are reported:<br>"I have recently tried to reduce the amount of food I throw away". % of strongly or moderately agree: 73.7%<br>"I always make a list of what I need before shopping. % of strongly or moderately agree: 68.5%<br><br>Mondejar-Jimenez did not report the frequencies.

	provided), factors related to food waste avoidance (e.g. save money, felling of guilt) [37]<br><br>Beliefs (constructs: environment, self-interest, and resources), general attitude and behavioral intention [38]		
**SFC as an Umbrella concept**	Environmental awareness and action, economical and effective options, and sustainable buying options. [39]<br><br>Demographics (gender, religion, academic year, love relationship and marital status, residence status, average expenditure in sustainable consumption behaviour). [10]<br><br>Demographic and socioeconomic characteristics, value orientation, pro-environmental worldview, and food-related environmental beliefs [6]<br>Knowledge, attitudes, demographic characteristics [8]<br><br>Social norms, ecological purchase behavior, and clusters based on demographic characteristics. [9]<br><br>Perceived moral intensity of climate change [15]<br><br>Demographic and socioeconomic characteristics, perceived barriers [40]<br><br>Religion: Muslim vs non-Muslim (Hindus and Buddhists), specific needs, convenience, intention, promotion/diffusion, governmental efforts [13]<br><br>Personality, attitudes, values, lifestyles, demographic and socio-economic characteristics. [12]<br>Emotional experience of shopping [11]	Sustainable consumption behavior in food [10,39]<br>[Food-related] environmental behaviors [6]<br>Pro-environmental behavior [in food] [8]<br>Sustainable food consumption behavior [9]<br>Climate-friendly food choices [15,40]<br>Green food consumption [13]<br>Purchase behaviour of sustainable food products [12]<br>Ethical food consumption [11]	The most frequently reported SFC behaviors were food waste prevention/avoidance and purchase/intake of local and seasonal products. An exception was “Eat food that has been grown locally” reported by 3.4 % of a sample of students in the US.<br><br>In Makiniemi and Vainio, 2013 “limit food waste” was the behavior with the highest mean score (5.1) out of 7 points, followed by “favor local food” (4.37), and “eat seasonal food” (4.35).<br><br>In Dopelt the most frequent reported behavior was “I buy food made in Israel” (4.01) and “I eat food according to the season” (3.30).<br><br>In Kamenidou, the highest score is for “Eat only seasonal fruits and vegetables” (5.46), and “Buy regional food products” (5.10), out of 7.<br><br>The least frequent behaviors were related to the reduction/moderation of meat consumption, with the avoidance of air transported products.

References of included articles

1. Akbar A, Ali S, Ahmad MA, Akbar M, Danish M. Understanding the Antecedents of Organic Food Consumption in Pakistan: Moderating Role of Food Neophobia. *Int. J. Environ. Res. Public Health Electron. Resour.* (2019) 16(20):22. doi: 10.3390/ijerph16204043

2. Dahm MJ, Samonte AV, Shows AR. Organic foods: do eco-friendly attitudes predict eco-friendly behaviors? *J. Am. Coll. Health* (2009) 58(3):195–202. doi: 10.1080/07448480903295292

3. Hamilton K, Hekmat S. Organic food and university students: a pilot study. *Nutr. Food Sci.* (2018) 48(2):218–27. doi: 10.1108/nfs-06-2017-0127

4. McReynolds K, Gillan W, Naquin M. An Examination of College Students' Knowledge, Perceptions, and Behaviors Regarding Organic Foods. *Am. J. Health Educ.* (2018) 49(1):48–55. doi: 10.1080/19325037.2017.1399837

5. Giampietri E, Bugin G, Trestini S. Exploring the interplay of risk attitude and organic food consumption. *Int. J. Food Syst. Dyn.* (2020) 11(3):189–201. doi: 10.18461/ijfsd.v11i3.49

6. Campbell-Arvai V. Food-related environmental beliefs and behaviours among university undergraduates A mixed-methods study. *Int. J. Sustain. High. Educ.* (2015) 16(3):279–95. doi: 10.1108/ijshe-06-2013-0071

7. Zámková M, Prokop M. Consumer behaviour of students when shopping for organic food in the Czech Republic. *Acta Univ. Agric. Silvic. Mendel. Brun.* (2013) 61(4):1191–201. doi: 10.11118/actaun201361041191

8. Dopelt K, Radon P, Davidovitch N. Environmental effects of the livestock industry: The relationship between knowledge, attitudes, and behavior among students in Israel. *Int. J. Environ. Res. Public. Health* (2019) 16(8):1359.

9. Kamenidou IC, Mamalis SA, Pavlidis S, Bara EZG. Segmenting the Generation Z Cohort University Students Based on Sustainable Food Consumption Behavior: A Preliminary Study. *Sustainability* (2019) 11(3):22. doi: 10.3390/su11030837

10. Anh PT, Lan NTN, Hanh NTM, Huy DTN, Loan BTT. Sustainable consumption behaviors of young people in the field of food and drinks: A case study. *J. Secur. Sustain. Issues* (2020) 9(May):36–47. doi: 10.9770/JSSI.2020.9.M(3)

11. Schoolman ED. Doing Right and Feeling Good: Ethical Food and the Shopping Experience. *Sociol. Perspect.* (2019) 62(5):668–90. doi: 10.1177/0731121419855980

12. Vecchio R, Annunziata A. Consumers' attitudes towards sustainable food: a cluster analysis of Italian university students. *New Medit* (2013) 12(2):47–55.

13. Mohd Suki N, Mohd Suki N. Does religion influence consumers' green food consumption? Some insights from Malaysia. *J. Consum. Mark.* (2015) 32(7):551–63. doi: 10.1108/JCM-02-2014-0877

14. Fernandez-Ferrin P, Bande B, Calvo-Turrientes A, Galan-Ladero MM. The Choice of Local Food Products by Young Consumers: The Importance of Public and Private Attributes. *Agribusiness* (2017) 33(1):70–84. doi: 10.1002/agr.21470

15. Makiniemi JP, Vainio A. Moral intensity and climate-friendly food choices. *Appetite* (2013) 66:54–61. doi: 10.1016/j.appet.2013.01.026

16. Diez JR, Antiguedad I, Agirre E, Rico A. Perceptions and Consumption of Bottled Water at the University of the Basque Country: Showcasing Tap Water as the Real Alternative towards a Water-Sustainable University. *Sustainability* (2018) 10(10):15. doi: 10.3390/su10103431

17. Barros KS, Bierhals IO, Assuncao MCF. Vegetarianism among first-year students at a public university in Southern Brazil, 2018. *Epidemiol. E Serv. Saude* (2020) 29(4):10. doi: 10.5123/s1679-49742020000400009

18. Forestell CA, Spaeth AM, Kane SA. To eat or not to eat red meat. A closer look at the relationship between restrained eating and vegetarianism in college females. *Appetite* (2012) 58(1):319–25. doi: 10.1016/j.appet.2011.10.015

19. Izmirli S, Phillips CJC. The relationship between student consumption of animal products and attitudes to animals in Europe and Asia. *Br. Food J.* (2011) 113(2–3):436–50. doi: 10.1108/00070701111116482

20. Kawasaki Y, Akamatsu R, Fujiwara Y, Omori M, Sugawara M, Yamazaki Y, et al. Is mindful eating sustainable and healthy? A focus on nutritional intake, food consumption, and plant-based dietary patterns among lean and normal-weight female university students in Japan. *Eat. Weight Disord. - Stud. Anorex. Bulim. Obes.* (2021). doi: 10.1007/s40519-020-01093-1

21. Llanaj E, Hanley-Cook GT. Adherence to healthy and sustainable diets is not differentiated by cost, but rather source of foods among young adults in Albania. *Br. J. Nutr.* (2020) :1–9. doi: 10.1017/S0007114520004390

22. Menozzi D, Sogari G, Veneziani M, Simoni E, Mora C. Eating novel foods: An application of the theory of planned behaviour to predict the consumption of an insect-based product. *Food Qual. Prefer.* (2017) 59:27–34. doi: 10.1016/j.foodqual.2017.02.001

23. Olfert MD, Barr ML, Mathews AE, Horacek TM, Riggsbee K, Zhou W, et al. Life of a vegetarian college student: Health, lifestyle, and environmental perceptions. *J. Am. Coll. Health* (2020) :1–8. doi: 10.1080/07448481.2020.1740231

24. Ruby MB, Alvarenga MS, Rozin P, Kirby TA, Richer E, Rutsztein G. Attitudes toward beef and vegetarians in Argentina, Brazil, France, and the USA. *Appetite* (2016) 96:546–54. doi: 10.1016/j.appet.2015.10.018

25. Suleiman AA, Alboqai OK, Kofahi S, Aughsteen AA, El Masri K. Vegetarianism among Jordan University students. *J. Biol. Sci.* (2009) 9(3):237–42. doi: 10.3923/jbs.2009.237.242

26. Vizcaino M, Ruehlman LS, Karoly P, Shilling K, Berardy A, Lines S, et al. A goal-systems perspective on plant-based eating: keys to successful adherence in university students. *Public Health Nutr.* (2021) 24(1):75–83. doi: 10.1017/S1368980020000695

27. Spencer EH, Elon LK, Frank E. Personal and professional correlates of US medical students' vegetarianism. *J. Am. Diet. Assoc.* (2007) 107(1):72–8. doi: 10.1016/j.jada.2006.10.034

28. Smith CF, Burke LE, Wing RR. Vegetarian and weight-loss diets among young adults. *Obes. Res.* (2000) 8(2):123–9. doi: 10.1038/oby.2000.13

29. Forleo M, Tamburro M, Mastronardi L, Giaccio V, Ripabelli G. Food consumption and eating habits: A segmentation of university students from central-south Italy. *New Medit* (2017) 16:56–65.

30. Pocol CB, Marinescu V, Amuza A, Cadar RL, Rodideal AA. Sustainable vs. Unsustainable Food Consumption Behaviour: A Study among Students from Romania, Bulgaria and Moldova. *Sustainability* (2020) 12(11):21. doi: 10.3390/su12114699

31. Al-Domi H, Al-Rawajfeh H, Aboyousif F, Yaghi S, Mashal R, Fakhoury J. Determining and addressing food plate waste in a group of students at the University of Jordan. *Pak. J. Nutr.* (2011) 10(9):871–8. doi: 10.3923/pjn.2011.871.878

32. Alattar MA, DeLaney J, Morse JL, Nielsen-Pincus M. Food waste knowledge, attitudes, and behavioral intentions among university students. *J. Agric. Food Syst. Community Dev.* (2020) 9(3):109–24. doi: 10.5304/jafscd.2020.093.004

33. Lorenz BA, Hartmann M, Langen N. What makes people leave their food? The interaction of personal and situational factors leading to plate leftovers in canteens. *Appetite* (2017) 116:45–56. doi: 10.1016/j.appet.2017.04.014

34. Mondejar-Jimenez JA, Ferrari G, Secondi L, Principato L. From the table to waste: An exploratory study on behaviour towards food waste of Spanish and Italian youths. *J. Clean. Prod.* (2016) 138:8–18. doi: 10.1016/j.jclepro.2016.06.018

35. Morata Verdugo MP, Gonzalez-Santana R, Blesa J, Frigola Canoves A, Esteve Mas MJ. [A study of the habits and food waste production of young university students]. *Nutr. Hosp.* (2020) 37(2):349–58. doi: 10.20960/nh.02833

36. Principato L, Secondi L, Pratesi CA. Reducing food waste: an investigation on the behaviour of Italian youths. *Br. Food J.* (2015) 117(2):731–48. doi: 10.1108/bfj-10-2013-0314

37. Wu Y, Tian X, Li X, Yuan H, Liu G. Characteristics, influencing factors, and environmental effects of plate waste at university canteens in Beijing, China. *Resour. Conserv. Recycl.* (2019) 149:151–9. doi: 10.1016/j.resconrec.2019.05.022

38. Lorenz BAS, Langen N, Hartmann M, Klink-Lehmann J. Decomposing attitudes towards food leftovers: Implications for general attitude, intention and behavior. *Br. Food J.* (2018) 120(11):2498–509. doi: 10.1108/bfj-08-2017-0430

39. Anh PT, Van Son D, Hong NTT, Huy DTN, Linh NNK. Factors affecting sustainable consumption choice in the field of food and drink: The case of university students in Hanoi. *Int. J. Entrep.* (2019) 23(1 Special Issue).

40. Makiniemi JP, Vainio A. Barriers to climate-friendly food choices among young adults in Finland. *Appetite* (2014) 74:12–9. doi: 10.1016/j.appet.2013.11.016
